# Multidetector computed tomography angiography of the renal arteries: normal anatomy and its variations*

**DOI:** 10.1590/0100-3984.2014.0048

**Published:** 2016

**Authors:** Carlos Fernando de Mello Júnior, Severino Aires Araujo Neto, Arlindo Monteiro de Carvalho Junior, Rafael Batista Rebouças, Gustavo Ramalho Pessoa Negromonte, Carollyne Dantas de Oliveira

**Affiliations:** 1 PhD, Adjunct Professor IV of Clinical Radiology, Universidade Federal da Paraíba (UFPB), João Pessoa, PB, Brazil.; 2 PhD, Adjunct Professor II of Clinical Radiology, Universidade Federal da Paraíba (UFPB), João Pessoa, PB, Brazil.; 3 PhD, Professor in the Department of Surgery, Universidade Federal da Paraíba (UFPB), João Pessoa, PB, Brazil.; 4 MD, Urologist, Professor of Urology, Faculdade de Ciências Médicas da Paraíba, João Pessoa, PB, Brazil.; 5 Medical Student, Universidade Federal da Paraíba (UFPB), João Pessoa, PB, Brazil.

**Keywords:** Anatomic variation, Renal artery, Multidetector computed tomography

## Abstract

Conventional angiography is still considered the gold standard for the study of the anatomy and of vascular diseases of the abdomen. However, the advent of multidetector computed tomography and techniques of digital image reconstruction has provided an alternative means of performing angiography, without the risks inherent to invasive angiographic examinations. Therefore, within the field of radiology, there is an ever-increasing demand for deeper knowledge of the anatomy of the regional vasculature and its variations. Variations in the renal vascular system are relatively prevalent in the venous and arterial vessels. For various conditions in which surgical planning is crucial to the success of the procedure, knowledge of this topic is important. The aim of this study was to familiarize the general radiologist with variations in the renal vascular system. To that end, we prepared a pictorial essay comprising multidetector computed tomography images obtained in a series of cases. We show patterns representative of the most common anatomical variations in the arterial blood supply to the kidneys, calling attention to the nomenclature, as well as to the clinical and surgical implications of such variations.

## Abstract (pt)

A angiografia convencional ainda é considerada o exame padrão ouro no estudo da anatomia e das doenças vasculares do abdome. Entretanto, com o advento da tomografia computadorizada com multidetectores e técnicas de reconstrução de imagens digitais, este exame tem-se tornado uma opção, com a vantagem de não ter os riscos habituais dos exames angiográficos invasivos. Com isso, o aprofundamento do conhecimento dos detalhes anatômicos da vasculatura regional e suas variações é cada vez mais exigido nesta área da radiologia. As variações do sistema vascular renal são relativamente prevalentes, tanto no leito venoso quanto no arterial. O conhecimento do tema é de importância nas várias condições em que o prévio planejamento cirúrgico é crucial para o sucesso do procedimento. Para familiarizar o radiologista geral, enriquecendo sua experiência sobre o tópico, os autores elaboraram um ensaio iconográfico a partir de uma série de casos extraídos do banco de imagens de tomografia computadorizada com multidetectores da região abdominal, com os padrões representativos das mais frequentes variações anatômicas da irrigação arterial renal, chamando a atenção para sua designação terminológica e para suas implicações clinicocirúrgicas.

## INTRODUCTION

Variations in the patterns of blood supply and ramification of the abdominal vessels are common in the vascular bed of the genitourinary system, and knowledge of these variations is almost as important as is that of the so-called "normal" pattern. Up until the middle of the last century, the vascular anatomy of the abdomen was a topic of discussion restricted to surgeons and anatomists. With the advent and rapid development of imaging tests, radiologists have become indispensible in the diagnostic process and therapy planning for many vascular conditions^(1)^. It is the job of the radiologist to identify and describe the anatomical patterns of venous and arterial vasculature, particularly when the tests in question are done in preparation for complex kidney surgeries.

Digital angiography continues to be the gold standard for comparison with any other type of tests for morphological analysis of renal arterial anatomy^(2,3)^. However, computed tomography angiography (CTA) studies carry fewer risks and are more accurate than digital angiography studies, with the advantage of evaluating not only the vascular lumina but also the vessel walls and other viscera, and is now used more frequently in various scenarios: kidney transplant, Takayasu's disease, and ureteropelvic junction (UPJ) stenosis due to compression of the inferior polar artery. In addition, CTA has the advantages of allowing a better evaluation of the renal collecting system-to identify hydronephrosis-and of the kidneys themselves-to identify tumors, parenchymal atrophy, and congenital disorders, such as horseshoe kidney and duplication of the renal pelvis^(4)^.

Digital image processing and manipulation in diagnostic workstations equipped with programs and monitors dedicated to this purpose are indispensible for CTA studies. Such studies allow two-dimensional (2D) and three-dimensional (3D) multiple reconstructions to be performed on the basis of raw data extracted from the original axial plane images. The multiplanar reconstruction (MPR), maximum intensity projection (MIP) and volume rendering (VR) methods are widely used and merit a brief explanation. The MPR method provides 2D sectional images in all axial, coronal, and sagittal planes, which can be perpendicular to the axial plane, but variations on oblique or even curved planes are particularly useful in the study of tortuous structures such as vessels. The MIP method selects the higher-density voxels in contiguous axial, coronal, or sagittal sectional images, summing and projecting them in a single image, generally in 3D. After intravenous injection of contrast, when vascular luminal density increases significantly, MIP highlights veins and arteries against the other less dense intra-abdominal structures. Combining consecutive sections allows long tortuous vascular segments, which usually enter and exit from an isolated conventional section plane, to be shown in a single image. This effect gives the MIP image the visual sensation of three dimensions. A limitation of this technique is in fact the excess of structures that are added to the image as the slab encompasses more sections, which may visually confuse the examiner. The VR method attributes opacity values ranging from 0% (transparent) to 100% (opaque) between various sections in any plane, in an artificial line of sight projection. By combining these values with luminous effects, the VR 3D image generated reproduces the perspective of depth in a more reliable way than does MIP^(1)^. Therefore, although MIP and VR have similar resolution and contrast, some authors, such as Urban et al.^(5)^, ascribe a certain advantage to VR, particularly in the visualization of tortuous vessels when it is necessary to determine which are closer or farther from the observer (more superficial or deeper in the examined region, for example).

The objective of this pictorial essay was to familiarize radiologists with the variations found in the renal vascular system, emphasizing prevalence, the most adequate appropriate terms and the clinical and surgical implications involved. To that end, we searched our teaching files, selecting sample cases in which multidetector computed tomography (MDCT) studies had produced scans that illustrated the most common anatomical patterns. The studies were carried out with a Brilliance 64-channel scanner (Philips, Eindhoven, the Netherlands). All patients were adults and were given intravenous iodinated contrast Ultravist^®^ (Bayer Pharma AG; Leverkusen, Germany), at a concentration of 769 mg/mL. The injection was applied with an injector pump (Envision CT; Medrad, Indianola, PA, USA) with a flow rate of 5 mL/s, and the dosage was approximately 1.5 mL/kg (maximum total dose of 150 mL). Only the post-contrast arterial phase was used for the sample cases. The axial sections (1 mm thick) were acquired at a pitch of 0.8, a reconstruction thickness of 2 mm, and a standard 250 mm field-of-view. During the arterial phase, image acquisition was started with a 6-s delay, after the threshold of 100 Hounsfield units had been reached at the region of interest within the abdominal aorta. The acquisition parameters used for the arterial phase in the abdominal protocol were similar to those used for CTA tests and showed sufficient spatial and temporal resolution to characterize the arterial vessels studied in this paper. The images were processed in a Philips Extended Brilliance workstation using a viewer program. Finally, we used the MPR (2D), MIP and VR (3D) methods to acquire images in the axial, coronal, and sagittal planes.

## NORMAL ANATOMY OF THE RENAL ARTERIES AND ITS VARIATIONS

The vascularization of the embryonic kidney (pronephros, mesonephros, and metanephros) originates from a group of lateral branches of the abdominal aorta. During the upward migration of the kidney to the lumbar region, many arterial branches regress and a main (or hilar) artery supplies blood to the renal parenchyma. Despite sequential regression of those structures, the caudal arteries, located between the tenth thoracic segment and the third lumbar segment, can persist in the fully formed kidney, evolving into the superior and inferior polar arteries^(1,6)^.

**Figure 1:**
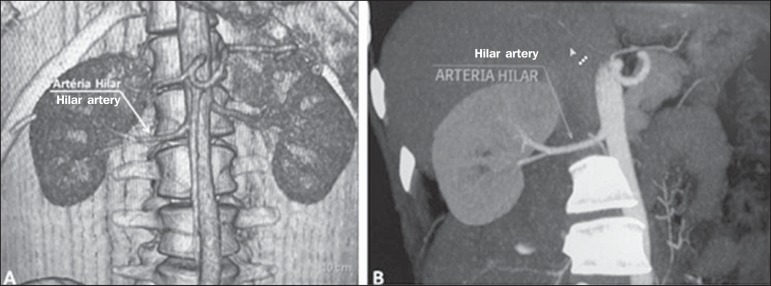
MDCT, frontal plane VR reconstruction (**A**) and coronal plane MIP reconstruction (**B**) showing the right hilar renal artery (arrow), which is a branch of the aorta that enters the kidney near the hilum and has terminal branches only at the hilum or renal sinus.

In the so-called "normal" pattern, the kidneys are supplied by a single main renal artery, which originates from the abdominal aorta at the L1-L2 level. The main renal artery is also referred to as the hilar artery, because it splits into two, three, or four branches near the hilum (Figure 1), providing the blood supply to various regions of the kidneys. In general, the hilar artery is 4-6 cm long and 5-6 mm in diameter. However, that classic configuration is seen in less than 25% of cases^(7)^.

Given the diverse patterns of renal arterial blood supply, a standardized nomenclature should be adopted, avoiding dubious terms and contemplating designations that carry an objective anatomical sense and are self-sufficient in transmitting an idea of the morphology to which they refer. Uniform usage of these terms among professionals in various fields is fundamental to facilitating communication, avoiding errors, and allowing statistical data from various studies to be synthesized. The terms "extra", "aberrant", and "supernumerary", which have been used by some authors^(8,9)^, should be avoided, because they minimize the importance of these vessels and do not have objective morphological meaning^(6,7)^. Despite the diversity and even disagreement among the various terms suggested, the nomenclature adopted by Sampaio et al.^(6)^ is, in our opinion, the one that comes closest to the aforementioned precepts and was therefore chosen to classify the findings described in this paper. The arterial patterns of the kidneys (normal and variants) are listed and described below, referenced to the corresponding examples in the figures.

**Hilar artery** (Figure 1) - Branch of the aorta that enters the kidney around the hilum and only at the hilum or renal sinus offers terminal branches. Palmieri et al. reported the prevalence of this pattern to be 62.49% in the right kidney and 72.50% in the left kidney^(7)^.

**Figure 2:**
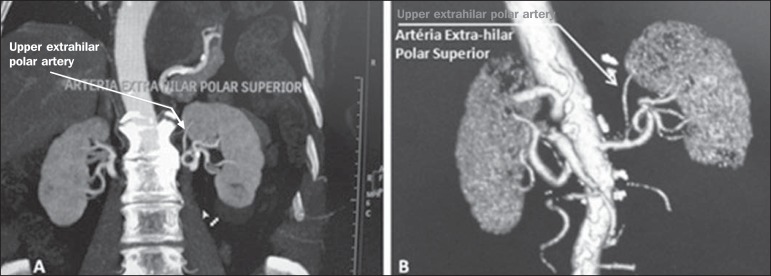
MDCT, coronal plane MIP reconstruction (**A**) and frontal plane VR reconstruction (**B**). The arrows indicate the left upper extrahilar polar artery, which branches off the left hilar artery and moves toward the left upper pole.

**Figure 3:**
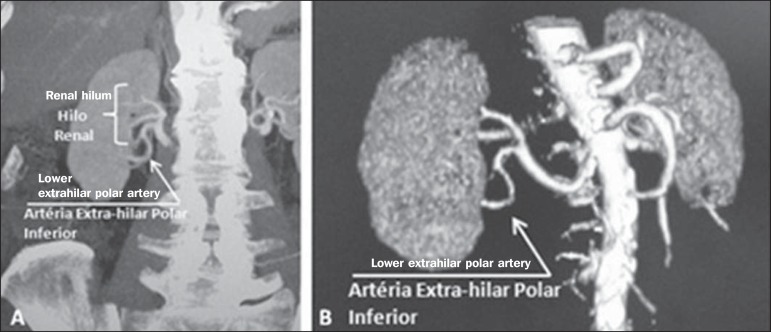
MDCT, coronal MIP plane reconstruction (**A**) and frontal plane VR reconstruction (**B**). The arrows indicate the right lower extrahilar polar artery, which branches off the right hilar artery and moves toward the right lower pole. The key shows the limits of the renal hilum.

**Upper and lower extrahilar artery** (Figures 2 and 3, respectively) - Branch originating from the hilar artery before it reaches the hilum and entering the renal parenchyma outside the hilum (at the upper or lower pole). The reported prevalence of an upper extrahilar artery is 28.6% and 11.6% in the right and left kidney, respectively, compared with and 0% and 1.4%, respectively, for that of a lower extrahilar artery.^(7)^

**Figure 4:**
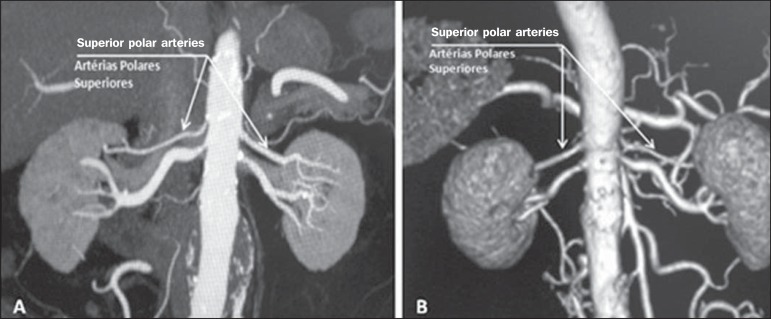
MDCT, coronal plane MIP reconstruction (**A**) and posterior plane VR reconstruction (**B**). The arrows indicate the right and left superior polar arteries, which branch off the aorta and move toward the right and left upper poles, respectively.

**Superior polar artery** (Figure 4) - Branch of the aorta that enters the kidney at the upper pole. The reported prevalence of a superior polar artery is 7.14% in the right kidney and 11.6% in the left kidney^(7)^.

**Inferior polar artery** - Branch of the aorta or of the common iliac artery that enters the kidney at the lower pole. The reported prevalence of an inferior polar artery is 3.57% in the right kidney and 2.9% in the left kidney^(7)^.

**Figure 5:**
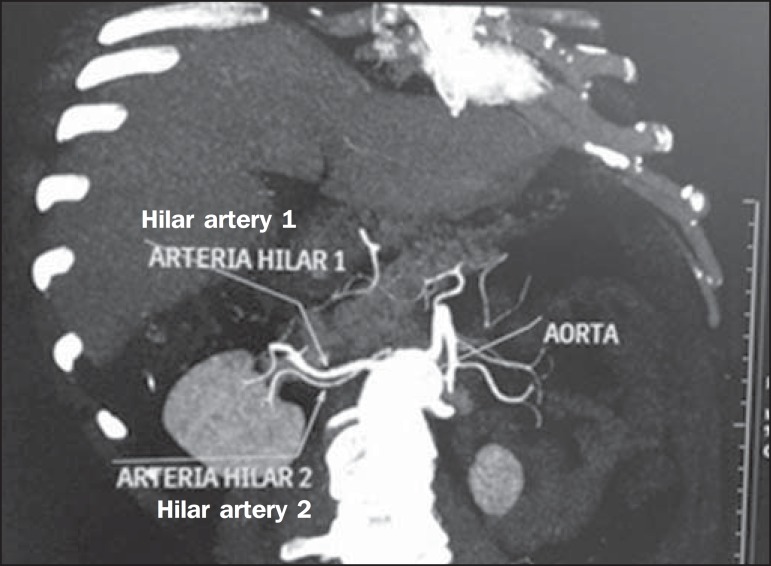
MDCT, oblique coronal plane MIP reconstruction. Note the right main hilar artery, designated hilar artery 1, and a right accessory hilar artery, designated hilar artery 2. In this case, both arteries originate from the aorta.

**Figure 6:**
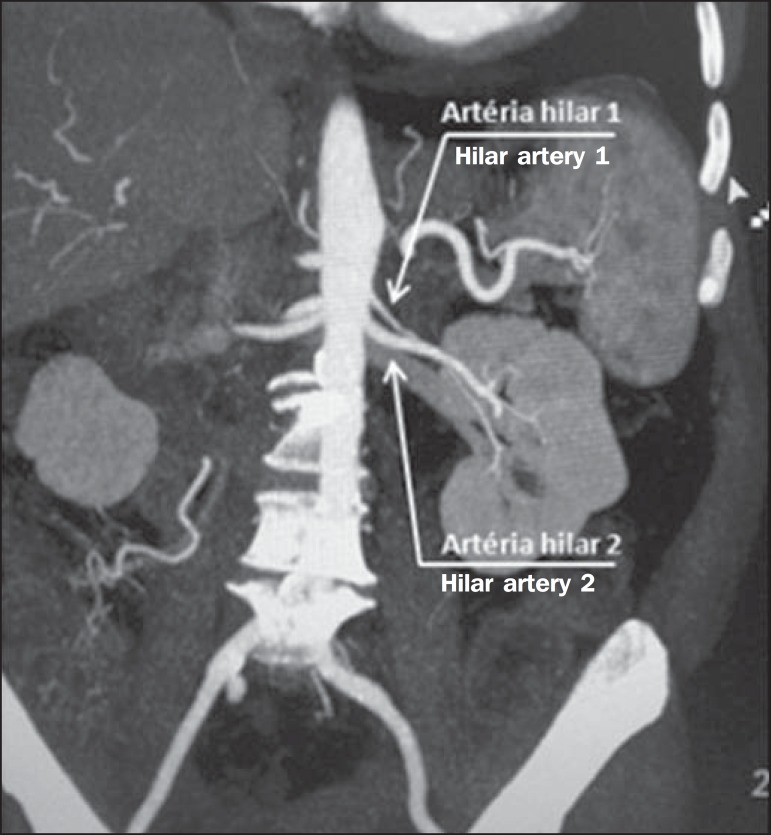
MDCT, coronal plane MIP reconstruction. Note the left main hilar artery (hilar artery 2) entering the hilum, as well as the left accessory hilar artery (hilar artery 1) that follows it. Both originate from the aorta.

**Early bifurcation** (Figures 5 and 6) - Right or left renal artery with a main trunk less than 1 cm long before branching. This pattern was observed in only one case out of 200 renal pedicles studied(^7^). In the case of two or more hilar arteries, the one with the largest caliber will be referred to as the main artery^(6)^.

The prevalence statistics for the morphological patterns mentioned above were restricted to the study by Palmieri et al.^(7)^, because the authors employed a nomenclature closest to that employed by Sampaio et al.^(6)^. The lack of uniformity in the nomenclature employed in many papers published on this subject hinders and many times precludes data unification or cross-study comparisons.

## CLINICAL AND SURGICAL IMPLICATIONS OF IMAGING FINDINGS

When planning surgical procedures such as partial nephrectomy, pyeloplasty for UPJ stenosis, and kidney transplantation, imaging studies are indispensible diagnostic tools^(1,3,5)^. The anatomical information provided can affect the chosen surgical technique.

Recent studies have shown that partial nephrectomy for tumors produces an oncological result equivalent to that of radical nephrectomy, with a lower rate of progression to chronic kidney disease and cardiovascular events^(10-12)^. When planning partial nephrectomies, previous knowledge of the vascular anatomy is indispensible. Reducing warm ischemia time is one of the technical measures that can improve the functional results of partial nephrectomy^(13-16)^. The main technique applied is segmental arterial clamping, which potentially improves renal function in the immediate postoperative phase, in comparison with clamping of the main artery^(17)^. The development of high-definition 3D models of renal vasculature based on imaging studies allows greater precision in the application of the segmental arterial clamping technique^(17)^.

Polar or extrahilar renal arteries are involved in 29-65% of cases of UPJ stenosis^(18,19)^. Prior knowledge of the presence of these vessels can influence the surgical approach because they can make endoscopic procedures more difficult and reduce the success rate of conventional treatment^(20-22)^. Therefore, MDCT offers advantages over methods such as ultrasound and intravenous urography in the evaluation of UPJ stenosis, particularly when applied in order to identify polar arteries.

**Figure 7:**
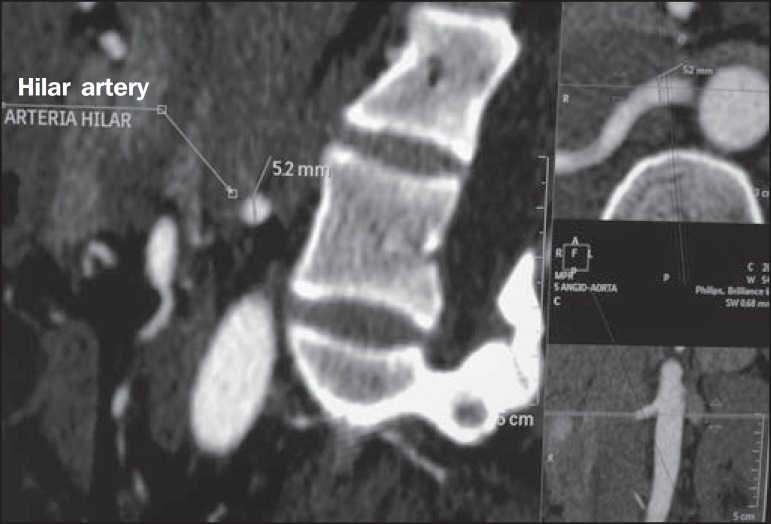
MDCT, oblique sagittal plane multiplanar reconstruction, for measuring the caliber of the right hilar artery. Axial and coronal plane images of the same region are shown in the right-hand margin. Note the orientation of the section, perpendicular to the vessel.

**Figure 8:**
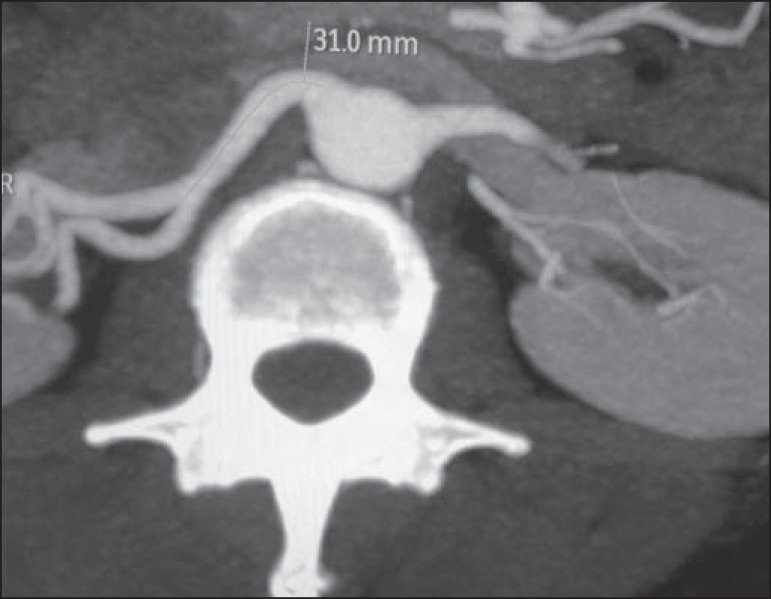
MDCT, axial plane MIP reconstruction, for measuring the length of the right hilar artery from its origin, in the aorta, to its first bifurcation.

In live-donor kidney transplantation, prior identification of a single renal artery is a favorable factor and lowers the incidence of complications. The presence of variations^(6)^ increases the incidence of vascular thrombosis, warm ischemia time, blood loss, and the difficulty of carrying out anastomosis, as well as the possibility of urinary fistulas and urethral lesions^(6,23)^. However, the rate of graft rejection in the first year and five-year survival rate do not seem to be affected by the presence of arterial anatomical variations^(23)^. As well as mentioning variations in quantity and bifurcation measurements, it is important that the radiology report makes reference to two other aspects: a) the orthogonal diameter of the renal arteries and its variations (Figure 7), given that anastomosis is difficult to perform in arteries with a diameter of less than 3 mm and there are greater risks of thrombosis; and b) the length of the artery from its origin to the first bifurcation (Figure 8), because surgeons recommend that the renal artery be at least 20 mm long to ensure good anastomosis^(24)^.

## PRACTICAL RECOMMENDATIONS IN COMPUTED TOMOGRAPHY ANGIOGRAPHY INTERPRETATION

Axial images are still the basis of diagnosis. However, MPR, MIP, and VR provide important additional information^(3)^. In CTA, it is recommended that the examiner operate the workstation directly. In practice, the dynamic use of reconstruction resources confers greater security and agility in comparison with the isolated use of films.

When interpreting and elaborating the test report, it is recommended that the radiologist follow a basic script. The identification of arterial calcifications is possible in the pre-contrast phase. During the arterial phase, both renal arteries should be followed, from emergence to the renal sinus, in order to identify early bifurcation and extrahilar branches, after which the kidneys should be checked for the presence of polar arteries. Especially in the case of kidney transplantation, it is important to mention the orthogonal diameter of the hilar arteries and occasional variations, as well as the length of the arteries from their origin to the first bifurcation.

## CONCLUSIONS

Variations in the arterial vasculature of the kidney are highly prevalent. The low risk and excellent accuracy of MDCT in the evaluation of the arterial anatomy of the kidney^(5)^ makes it a practicable alternative to digital angiography in many situations. Considering the importance of this anatomy in the treatment planning for various clinical and surgical urological conditions, the radiologist should be prepared to identify and describe findings using standardized terminology that is in accordance with the consensus in the literature.
